# Mesh Exposure and Associated Risk Factors in Women Undergoing Transvaginal Prolapse Repair with Mesh

**DOI:** 10.1155/2013/926313

**Published:** 2013-09-08

**Authors:** Elizabeth A. Frankman, Marianna Alperin, Gary Sutkin, Leslie Meyn, Halina M. Zyczynski

**Affiliations:** ^1^Division of Urogynecology, Department of Obstetrics, Gynecology and Reproductive Sciences, Magee-Womens Hospital, University of Pittsburgh School of Medicine, 300 Halket Street, Pittsburgh, PA 15213, USA; ^2^Magee-Womens Research Institute, 204 Craft Avenue, Pittsburgh, PA 15213, USA

## Abstract

*Objective*. To determine frequency, rate, and risk factors associated with mesh exposure in women undergoing transvaginal prolapse repair with polypropylene mesh. *Methods*. Retrospective chart review was performed for all women who underwent Prolift Pelvic Floor Repair System (Gynecare, Somerville, NJ) between September 2005 and September 2008. Multivariable logistic regression was performed to identify risk factors for mesh exposure. *Results*. 201 women underwent Prolift. Mesh exposure occurred in 12% (24/201). Median time to mesh exposure was 62 days (range: 10–372). When mesh was placed in the anterior compartment, the frequency of mesh exposure was higher than that when mesh was placed in the posterior compartment (8.7% versus 2.9%, *P* = 0.04). Independent risk factors for mesh exposure were diabetes (AOR = 7.7, 95% CI 1.6–37.6; *P* = 0.01) and surgeon (AOR = 7.3, 95% CI 1.9–28.6; *P* = 0.004). *Conclusion*. Women with diabetes have a 7-fold increased risk for mesh exposure after transvaginal prolapse repair using Prolift. The variable rate of mesh exposure amongst surgeons may be related to technique. The anterior vaginal wall may be at higher risk of mesh exposure as compared to the posterior vaginal wall.

## 1. Introduction

 Mesh augmentation has been widely adopted for pelvic floor reconstructive procedures [1]. Sacral colpopexy is considered to be the “gold standard” surgical procedure based on favorable subjective and objective outcomes and a relatively low complication rate [2–4]. It has demonstrated superior durability when compared to transvaginal plication techniques using native tissues [2, 5].

In 2004, the Food and Drug Administration (FDA) approved the first commercial “system” or “kit” for the transvaginal delivery of polypropylene mesh into the vesicovaginal and/or rectovaginal plane for the treatment of uterine or vaginal vault prolapse. The goal of transvaginal mesh augmentation was to provide the durability of sacral colpopexy while avoiding the morbidity associated with laparotomy or prolonged laparoscopy. Following FDA approval, transvaginal mesh kits were widely adopted with an estimated 75,000 transvaginal mesh procedures for prolapse performed in 2010 [6].

In 2008 and 2011, the FDA issued statements due to concern regarding the frequency of complications associated with the use of transvaginal mesh for prolapse repair [6, 7]. Mesh exposure appears to be the most common complication and is documented by the visualization of graft material within the vagina. Although this public advisory resulted in the removal of several mesh kits from the market, investigation of clinical outcomes following transvaginal mesh placement affords an opportunity for improvement upon the technology or technique employed.

Mesh exposure is a known complication following any graft placement and has been reported in 0–6.4% of patients after abdominal sacral colpopexy [4, 8]. The variable frequency of exposure appears to be influenced by selection or choice of graft material [4]. Risk factors for mesh exposure after sacral colpopexy include use of expanded polytetrafluoroethylene mesh, concurrent hysterectomy, and smoking [9].

Less is known about the frequency and risk factors for mesh exposure after transvaginal polypropylene mesh placement. Widely varied frequencies of mesh exposure following Prolift Pelvic Floor Repair System (Gynecare, Somerville, NJ) have been reported, ranging from 1.6% to 19% [10–16]. Risk factors include concurrent hysterectomy and use of an inverted T colpotomy [13]. Mesh exposure following Prolift is the most common procedure-related complication and may require additional surgical intervention (in-office or operating room) [10–14]. Frequency and risk factors associated with mesh exposure after transvaginal mesh augmentation may influence surgical counseling and choice of reconstructive procedure.

The primary objective of our study was to determine the frequency and rate of mesh exposure in women who underwent transvaginal prolapse repair with polypropylene mesh using Prolift Pelvic Floor Repair System at a single institution. Secondarily, we sought to determine demographic, clinical, and surgical risk factors associated with mesh exposure.

## 2. Materials and Methods

 After obtaining University of Pittsburgh Institutional Review Board approval, a retrospective analysis of medical records for all women who underwent Prolift Pelvic Floor Repair System (Gynecare, Somerville, NJ) at Magee-Womens Hospital, Pittsburgh, Pennsylvania, between September 2005 and September 2008 was performed. The attending staff within the Division of Urogynecology consisted of five experienced gynecologic surgeons, three of whom completed an FPMRS fellowship. The remaining two had 10–20 years of vaginal surgery experience. Each surgeon was proficient in related procedures such as sacrospinous ligament colpopexy, vaginal paravaginal repairs, and transobturator midurethral slings before attending an extramural intraoperative preceptorship on Prolift placement. All cases involved FPMRS fellows' participation. We previously found that fellows' participation did not increase the perioperative morbidity [14]. The case logs for each of the five staff surgeons were used to identify all women who underwent Prolift during the study interval. Cases performed by these surgeons at other institutions or without postoperative followup at the Women's Center for Bladder and Pelvic Health of Magee-Womens Hospital were excluded.

The total and posterior Prolift procedures were performed according to the manufacturer's Instructions for Use (IFU) guide [17]. Anterior Prolift grafts were placed with a modification that involved a supplementary apical anchoring of the mesh to both sacrospinous ligaments for women with anterior and apical support defects who did not require mesh reinforcement of the posterior compartment. This apical modification of the anterior Prolift graft has been previously described by Alperin et al. [14]. Anterior Prolift without modification was performed early in our surgical experience prior to the adoption of the apical modification technique.

Inpatient and outpatient medical records were reviewed for each patient. Relevant demographic, medical, and surgical variables were collected. Pelvic organ prolapse was quantified and staged according to International Continence Society definitions [18]. Mesh exposure was defined as mesh visible within the vagina on postoperative speculum examination. The location of the mesh exposure was based on the examining physician's description. Overall compartment-specific mesh exposure frequency was calculated for the anterior and posterior compartments using the number of mesh exposures in the compartment as the numerator and total number of procedures with mesh placed in that compartment as the denominator. A separate analysis of mesh exposure frequency was performed for total Prolift procedures. Resolution of mesh exposure was defined as the absence of previously visible mesh on speculum examination. Postoperative examinations were performed by the patient's surgeon or another attending urogynecologist.

Results are presented as mean ± standard deviation (SD) or median (range) for continuous variables. Categorical variables are presented as percentages. Women with mesh exposure were compared to women without mesh exposure. Hypothesizing that early mesh exposure may be due to incision dehiscence and late exposure may be secondary to avascular necrosis of the vaginal wall, we compared characteristics of women with early mesh exposure (≤42 days) to those with late (>42 days) exposure. We dichotomized mesh exposures as early or late based upon their identification at the 6-week post-op visit (42 days). This is consistent with the window for perioperative (early) morbidity in the Clavien-Dindo classification system [19].

 Demographic, clinical, and surgical variables were compared between groups using Student's *t*-test or Fisher's exact test, where appropriate. Univariable analysis was performed prior to the development of a multivariable model in order to identify variables associated with mesh exposure. Multivariable logistic regression was performed to identify independent risk factors for mesh exposure. Differences in the frequency of mesh exposure between the anterior and posterior compartments were evaluated using the binomial test of proportions among all Prolift procedures and McNemar's test among total Prolift procedures. Statistical analyses were performed using SPSS (version 17.0; SPSS Inc., Chicago, IL). Statistical significance was evaluated at the two-sided 0.05 significance level.

## 3. Results

Mean age of the 201 women who underwent Prolift was 66 ± 8 (mean ± SD) years. Most women were Caucasian (90%) and postmenopausal (96%) and had undergone a hysterectomy previously (61%). Preoperatively, 17% of women had stage II prolapse, 75% had stage III prolapse, and 8% had stage IV prolapse. Mesh augmented suspension of the anterior and posterior vaginal walls was most often performed utilizing the Total Prolift system (107/201, 53%). Isolated anterior Prolift grafts were performed per manufacturer's IFU in 5/201 (2%) and the anterior Prolift with apical modification was utilized in 60/201 (30%). The remainder of cases were discrete posterior Prolift (29/201, 15%). Median duration of followup was 339 days (interquartile range: 148–438). Median number of postoperative visits was 5 (range: 1–14).

Mesh exposure occurred in 12% (24/201). The rate of mesh exposure was 14 per 100 woman-years. Median time to mesh exposure was 62 days (range: 10–372). Selected demographic, clinical, and surgical variables of the women with and without mesh exposure are described in Table 1. Univariable analysis identified a greater change in hemoglobin (preoperative minus postoperative hemoglobin; *P* = 0.05) and more women with diabetes (*P* = 0.03) among women with mesh exposure. There were no other statistically significant differences between women with and without mesh exposure, including history of or current smoking (*P* = 0.30), prior (*P* = 0.27) or concomitant hysterectomy (*P* > 0.99), and prior incontinence or prolapse procedures (*P* = 0.26 and 0.81, resp.).

Multivariable regression analysis identified the following independent risk factors for mesh exposure: diabetes (AOR = 7.7,  95% CI 1.6–37.6;  *P* = 0.01) and surgeon (AOR = 7.3,  95% CI 1.9–28.6;  *P* = 0.004). When the first half of each surgeon's case load was compared to the second half, there was no difference in the frequency of mesh exposure (*P* = 0.09).

Early mesh exposure was noted in 5/24 (21%) women and late in 19/24 (79%) women. When early and late exposures were compared, there were no differences between smoking, diabetes, change in hemoglobin, or surgeon (*P* > 0.05).

Mesh exposure was limited to 1 compartment in all affected women. There were 4 apical mesh exposures noted. All occurred in women who had undergone total Prolift. Anterior vaginal wall mesh exposure was observed in 15 women (total Prolift 7/15, anterior Prolift with modification 8/15). Posterior vaginal wall mesh exposure was noted in 4 women (total Prolift 3/4, posterior Prolift 1/4). Mesh exposure location was not documented in 1 woman who had undergone total Prolift.

When an overall compartment-specific mesh exposure frequency was calculated, the frequency of mesh exposure was higher in the anterior versus posterior compartment (15/172, 8.7% versus 4/136, 2.9%; *P* = 0.04). In cases where both the anterior and posterior compartments were at risk (total Prolift), the higher frequency of mesh exposure in the anterior compartment did not reach statistical significance when compared to the frequency of mesh exposure in the posterior compartment (7/106, 6.6% versus 3/106, 2.8%; *P* = 0.34).

All patients with mesh exposure were treated with vaginal estrogen and mesh exposure resolved in 3 cases with this conservative treatment. Mesh excision was performed in the outpatient office setting and/or in the operating room in 18/24 (75%) (Table 2).

 Visceral injuries occurred in 4/201 (2%). Trocar-related injuries to the bladder occurred in 2/201 (1%) of women. Cystotomy occurred at the time of vaginal dissection in 2 women. In both cases of cystotomy, the bladder was repaired primarily in 2 layers. The surgeon elected to place a biologic graft (Surgisis, Cook, West Lafayette, IN) over the repair prior to proceeding with placement of the polypropylene mesh contained in the Prolift kit. Rectal injury occurred in 2 cases; one was at the time of vaginal dissection, and the second was caused by blunt retraction. In both cases, the primary repair was followed by the Prolift graft placement. Since both injuries were distal to the edge of the posterior mesh and there would be no mesh overlying the primary repair site, the decision was made to proceed with mesh placement. No further complications were encountered in these two subjects. Intraoperative or postoperative hemorrhage requiring blood transfusion occurred in 5/201 (2.5%).

## 4. Discussion

The French tension-free vaginal mesh (TVM) group, the consortium of surgeons who developed the trocar-based polypropylene mesh procedure which predated the commercially available Prolift Pelvic Floor Repair System, has proposed detailing the frequency of “vaginal exposition” in addition to “infection” and “periprosthetic retraction” as three primary groups of complications [20]. We report a 12% frequency of mesh exposure following transvaginal polypropylene mesh insertion using the Prolift Pelvic Floor Repair System with a median duration of followup of 339 days. This frequency is similar to the data reported by the TVM group [13] (12.3%) but higher than that reported by other authors [10, 11, 14]. The longer duration of followup in our study as compared to other authors (48 weeks versus 8–12 weeks) may contribute to a higher but more accurate frequency of mesh exposure.

Complications following transvaginal mesh placement may be related to the technology utilized (i.e., mesh and/or trocar delivery system), the surgical technique (i.e., depth of dissection and tissue handling), or patient characteristics. Given that polypropylene mesh has been used successfully in abdominal pelvic reconstructive procedures with a low frequency of exposure [4, 8], it seems less likely that the higher frequency of mesh exposure associated with Prolift is related to the graft material itself. Rather, our data suggest that the trocar-based delivery system, surgical technique (employment of vaginal incisions), and individual patient characteristics may have a greater influence on mesh exposure risk.

The variation in mesh exposure frequency reported by different authors and the variation in exposure frequency between surgeons in our study suggest that surgical technique plays an important role in mesh exposure. Since mesh exposure frequency did not change when the first half of each surgeon's case load was compared to the second half, our data suggest that surgeons' outcomes were independent of their surgical volume and may reflect the depth of dissection. Full-thickness dissection of the vaginal epithelium, as described by Fatton et al. [10], is required to minimize tissue necrosis. We hypothesize that split-thickness vaginal dissection may disrupt the blood supply of the vaginal epithelium, either impeding tissue healing or contributing to avascular necrosis. Suture pull-through and dehiscence of the incision may occur if the sutures used to close the vaginal incision(s) are not placed at an adequate distance from the edge of the vaginal incision, particularly if full-thickness dissection has not been achieved.

Procedure-related blood loss is related to surgical technique, patient's anatomy, and the trocar-based mesh delivery system. Reports of hemorrhage and clinically significant postoperative hematomas after Prolift can be found in the literature [21–25]. Although intraoperative hemorrhage immediately following the passage of a trocar is nearly certainly related to laceration of a large vascular bundle, whether postoperative hematoma formation is due to the dissection or trocar passage is unknown. We observed a trend toward greater change in hemoglobin (preoperative minus postoperative hemoglobin) associated with mesh exposure but no difference in estimated intraoperative blood loss. Since estimation of blood loss is often inaccurate, it is possible that the lack of difference in estimated intraoperative blood loss between groups is due to inaccurate quantification by the surgeon [26]. We, however, believe that the trend toward greater change in hemoglobin among women with mesh exposure serves as a proxy for postoperative retroperitoneal bleeding and possible hematoma formation. Although no definitive data exist, it appears that a hematoma in the vaginal wall interferes with normal healing and may increase the risk of mesh exposure. Unlike intraperitoneal dissection of the vesicovaginal plane where capillary or serous oozing would decompress into the peritoneal cavity, in the case of extraperitoneal vaginal dissection, the incision line is the easiest route of drainage of a postoperative hematoma. If the vaginal epithelium has been devitalized during the vaginal dissection, incision breakdown may occur more often if a postoperative hematoma or seroma places additional tension on the suture line. For this reason, we emphasize the importance of excellent hemostasis throughout the procedure. A randomized controlled trial comparing trocar-based transvaginal mesh delivery systems to systems which do not utilize trocars would generate information regarding the role of trocars in blood loss and hematoma formation.

 Patient characteristics also influence the risk of mesh exposure. We observed a 7-fold increased risk of mesh exposure following Prolift in women with diabetes. The microvascular changes associated with diabetes are a known inhibitor of wound healing and perioperative hyperglycemia has been associated with an increased risk of surgical site infection [27]. These factors may explain the higher frequency of mesh exposure observed amongst women with diabetes. Future prospective studies will allow collection of data regarding glycemic control in diabetic women undergoing Prolift to determine whether mesh exposure risk is lessened in patients with optimal blood sugar control.

 Concurrent hysterectomy has previously been shown to be a risk factor for mesh exposure after Prolift [13]. Although we were unable to confirm this finding, the lack of association may be due to small proportion of women (19/201) in our cohort undergoing hysterectomy at the time of transvaginal mesh procedure.

 It is unclear whether the etiology of an early (≤42 days) mesh exposure differs from a late (>42 days) mesh exposure. We suspect that early mesh exposures are more likely related to the dehiscence of the vaginal incision(s) and late exposures are due to tissue necrosis. However, due to the relatively small number of early mesh exposures (5/24) and the limitations of the data regarding the proximity of the mesh exposure location to surgical incisions, we are unable to correlate clinical and surgical variables with early and late mesh exposures. Prospective acquisition of this data along with a larger sample may assist in determining independent risk factors and temporal associations for early and late mesh exposures. Histologic data from tissue biopsies of women with and without mesh exposure may also be helpful in determining etiology.

 We observed that mesh exposure occurs more frequently in the anterior compartment. Although the compartment-specific mesh exposure frequency was not significantly different when total Prolift procedures only were considered, this finding may be due to small sample size and low frequency of mesh exposure. It is unknown why the anterior compartment may be more vulnerable to mesh exposure. One of the factors that may play a role in the different rates of mesh exposure in anterior versus posterior compartments is a potentially differential tissue-implant interface in these compartments [28]. A mismatch between biomechanical properties of the implant and the surrounding tissue interferes with the appropriate load transmission and may contribute to an increased risk of mesh exposure in the anterior vaginal wall. The anterior Prolift mesh is larger than the posterior portion. The larger mesh load with anterior Prolift may play a role in the increased rate of mesh exposures in the anterior compartment. It is also possible that the blood supply to the anterior vagina may be less as compared to the posterior vaginal wall, placing the anterior wall at risk for inadequate wound healing and mesh exposure. Future studies should attempt to identify biologic characteristics and biomechanical properties that contribute to compartment-specific mesh exposure risk.

It has been proposed that surgical technique to limit mesh exposure may result in a higher frequency of visceral injuries. That is, in order to obtain full-thickness dissection of the vaginal epithelium to decrease the risk of mesh exposure, the surgeon must risk injury to the bladder or bowel, resulting in a greater incidence of cystotomy, enterotomy, or proctotomy. We observed a 2% frequency of visceral injury (excluding trocar injuries) in the setting of a 12% mesh exposure frequency. These data are similar to the frequencies reported by Collinet et al. [13] (1.8% visceral injury and 12.3% mesh exposure frequency) for the TVM group. Furthermore, a randomized controlled trial of anterior and posterior colporrhaphy with or without mesh augmentation resulted in a higher frequency of visceral injury in women undergoing traditional colporrhaphy (2.9% when no mesh was placed versus 0% when mesh augmentation was performed), suggesting that full-thickness dissection of the vaginal epithelium for appropriate vaginal mesh placement does not increase the risk of bladder or bowel injury at the time of surgery [29].

Our study is strengthened by the large number of women in the cohort with a median followup of 339 days and 5 visits. However, several biases exist which limit our findings. Since follow-up examinations were performed by the operating surgeon, mesh exposure reporting bias may exist. Future studies will be strengthened by the use of an independent examiner. Although all women who have undergone Prolift since the adoption of this procedure at our institution were included in the analyses, selection bias may exist since women were not randomized to this procedure. There are numerous commercially available transvaginal mesh “kits” available for use. These “kits” differ in mesh characteristics, mesh load, and delivery system. As a result, our findings may only be applied to those women undergoing Prolift Pelvic Floor Repair System. Lastly, our institutional modification of the anchoring points of the mesh in the anterior Prolift kit to include sacrospinous attachments limits our ability to compare anterior compartment mesh exposures for this specific system to other published studies of Prolift. However, the extent of anterior vaginal wall dissection was not greater leading us to conclude that the modification most likely does not contribute to the overall mesh exposure risk.

 Mesh exposure is a known complication of any procedure utilizing synthetic graft material. Transvaginal prolapse repair utilizing the Prolift Pelvic Floor Repair System is associated with a 12% mesh exposure frequency. Women with diabetes should be counseled that they have a 7-fold increased risk of mesh exposure. When selecting the compartment(s) for mesh augmentation, surgeons should be aware that the anterior compartment may be at higher risk of mesh exposure as compared to the posterior compartment.

## Figures and Tables

**Table 1:** Selected demographic, clinical, and surgical variables (*N* = 201).

Characteristic	Mesh exposure (*N* = 24)	No mesh exposure (*N* = 177)	*P *
Age (years)*	67.3 ± 8.8	66.1 ± 8.1	0.49
Body Mass Index (kg/m^2^)*	28.2 ± 4.0	27.9 ± 5.0	0.78
Smoking status			0.30
Nonsmoker	14/23 (61%)	119/173 (69%)	
Current smoker	2/23 (9%)	6/173 (3%)	
History of smoking	7/23 (30%)	48/173 (28%)	
Medical comorbidities			
Diabetes	4/23 (17%)	7/164 (4%)	0.03
Hypertension	12/24 (50%)	99/175 (57%)	0.66
Surgical history			
Prior incontinence procedure	2/24 (8%)	34/174 (20%)	0.26
Prior prolapse procedure	6/24 (25%)	53/176 (30%)	0.81
Prior hysterectomy	12/24 (50%)	111/177 (63%)	0.27
POPQ measurements			
Preoperative Ba (cm)*	+3.0 ± 3.0	+2.3 ± 2.6	0.23
Preoperative Bp (cm)*	+0.9 ± 3.7	+0.7 ± 3.1	0.81
Preoperative C (cm)*	−0.7 ± 5.0	−1.7 ± 4.4	0.30
Preoperative TVL (cm)*	8.6 ± 1.2	8.5 ± 2.2	0.77
Surgical variables			
Concomitant hysterectomy	2/24 (8%)	17/176 (10%)	>0.99
EBL (ccs)*	171 ± 194	140 ± 101	0.45
Δ hemoglobin (preoperative – postoperative in g)*	2.9 ± 1.2	2.4 ± 1.1	0.05

*Mean ± SD.

**Table 2:** Management and outcomes of mesh exposure (*N* = 24).

	Estrogen cream alone (*N* = 6)	Mesh excision in the office* (*N* = 9)	Mesh excision in operating room* (*N* = 5)	Mesh excision in operating room and office* (*N* = 4)
Resolved	3	2	3	4
Not resolved	2	4	1	0
Unknown^†^	1	3	1	0

*All patients undergoing office and/or operating room mesh excision also used estrogen cream.

^†^No speculum examination since mesh exposure documented or since excision.
